# A Microfluidic Immunostaining System Enables Quality Assured and Standardized Immunohistochemical Biomarker Analysis

**DOI:** 10.1038/srep45968

**Published:** 2017-04-05

**Authors:** Seyong Kwon, Chang Hyun Cho, Youngmee Kwon, Eun Sook Lee, Je-Kyun Park

**Affiliations:** 1 Department of Bio and Brain Engineering, Korea Advanced Institute of Science and Technology (KAIST), 291 Daehak-ro, Yuseong-gu, Daejeon 34141, Republic of Korea; 2 Center for Breast Cancer, National Cancer Center, 323 Ilsan-ro, Ilsandong-gu, Goyang-si, Gyeonggi-do 10408, Republic of Korea; 3 KAIST Institute for Health Science and Technology, 291 Daehak-ro, Yuseong-gu, Daejeon 34141, Republic of Korea

## Abstract

Immunohistochemistry (IHC) plays an important role in biomarker-driven cancer therapy. Although there has been a high demand for standardized and quality assured IHC, it has rarely been achieved due to the complexity of IHC testing and the subjective validation-based process flow of IHC quality control. We present here a microfluidic immunostaining system for the standardization of IHC by creating a microfluidic linearly graded antibody (Ab)-staining device and a reference cell microarray. Unlike conventional efforts, our system deals primarily with the screening of biomarker staining conditions for quantitative quality assurance testing in IHC. We characterized the microfluidic matching of Ab staining intensity using three HER2 Abs produced by different manufacturers. The quality of HER2 Ab was also validated using tissues of breast cancer patients, demonstrating that our system is an efficient and powerful tool for the standardization and quality assurance of IHC.

Molecular-targeted cancer therapy has shown its effectiveness in treatments such as tamoxifen (breast), sorafenib (liver and kidney), trastuzumab (breast and stomach), and bevacizumab (brain, cervical, colorectal, kidney, and lung)^1^,^2^,^3^,^4^. As a consequence of the beneficial effects of these drugs on cancer survival rates, various kinds of molecules associated with cancer development have been studied and targeted for drug development, including hormone receptors, signal transduction molecules, and cell surface molecules^5^,^6^,^7^,^8^.

Biomarker-based cancer diagnosis techniques, such as immunohistochemistry (IHC), are essential to obtain the therapeutic benefits of these drugs. For example, the positive expression of estrogen receptors (ER) predicts the effect of tamoxifen in breast cancer treatment, and trastuzumab improves overall survival in the early stage of human epidermal growth factor receptor 2 (HER2)-expressing breast cancer. Commensurate with the emergence of several new drugs to combat cancers, the number of biomarkers for identifying cancer types has increased exponentially^9^,^10^. To validate new drugs and biomarkers, large-scale inter-laboratory and inter-hospital cooperative clinical studies have become critical^11^,^12^. However, with widespread use of IHC in cancer diagnosis and research, test quality assurance and standardization have been highlighted as major issues in these fields^13^,^14^. The IHC process is difficult to standardize, because immunostaining quality is highly variable among laboratories, technicians, and protocols^15^. Nevertheless, there is a high demand for quality assurance and standardization of IHC, due to IHC being among the primary diagnostic tests for cancers^16^,^17^,^18^.

For these reasons, a number of researchers and national agencies concerned with cancer healthcare have tried to standardize IHC protocols. For example, the American Society of Clinical Oncology/College of American Pathologists (ASCO-CAP) suggested guidelines for the effective use of the well-known breast cancer drug trastuzumab, using improved HER2 clinical test standards^19^,^20^. To improve the reproducibility of IHC testing, a fully-automated IHC machine has been developed that can handle all of the steps in the IHC process^21^,^22^. Although this technology can ensure the quality and reproducibility of tests, the quality of reagents, including antibodies (Abs), cannot be controlled by these machines.

The quality assurance of immunostaining in hospitals or laboratories begins with the reproducibility of protein staining and the control of non-specific binding of Abs^23^. Usually, hospital pathology laboratories prepare positive/negative reference samples for major cancer biomarkers, such as cell blocks^24^. When a new Ab is introduced to the laboratory, an arbitrary concentration of Ab is selected for the test staining. After comparison of staining intensity with that of the preserved positive/negative reference samples previously immunostained with Ab, the Ab test staining is repeated using a newly introduced Ab, the concentration or incubation time being varied until the staining intensity approaches that of the reference (Fig. 1A)^25^,^26^. However, reproducibility cannot be assured with this conventional quality control system due to the qualitative nature of the staining intensity validation in the process flow. Moreover, this process consumes significant time and resources, considering the short reagent storage time and variable quality of Abs.

To address this problem, we first developed a microfluidic immunostaining system to incubate linearly graded concentrations of Abs on a reference sample, for quantitative standardization of staining intensity and verification of Ab reagent quality (Fig. 1B). A newly designed linear gradient generator was employed for the robust use of the complex microfluidic system. The system requires only 10 min to screen 2 h Ab incubation results at various Ab concentrations. We were able to simultaneously incubate eight linearly graded concentrations of Ab with microfluidic channels on one reference sample. In this work, breast cancer tissues were employed to demonstrate that our system is appropriate for clinical use in standardized HER2 testing. Three HER2 Abs from different Ab manufacturers were standardized at one reference intensity using SKBR3 cell section slides. An Ab incubation condition, which avoids non-specific binding of quantum dot conjugated IgG (QD-IgG), was also demonstrated using our system. Additionally, we verified the HER2 Ab quality using a cell slide containing both positive and negative references of HER2. We believe that the HER2 Ab quality verification conducted using our system can directly reflect IHC staining quality.

## Results

### Microfluidic immunostaining system

**Figure 1:**
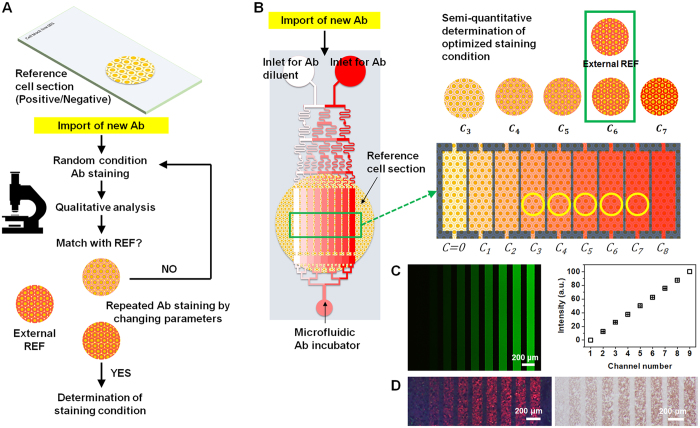
Microfluidic immunostaining system for antibody (Ab) quality verification and standardization. (**A**) Conventional immunostaining standardization process flow. A biomarker-specific cell or tissue sample that stained with optimized intensity is stored as a reference. When a new Ab is introduced, repetitive immunostaining is performed to subjectively match the staining intensity to the reference. (**B**) A positive control cell block section is immunostained with linearly graded concentrations of Ab. The staining intensity of the reference is compared with those resulting from Ab incubation at various Ab concentrations to identify the best match. (**C**) Images of the microchannels and cell sections resulting from the concentration gradient generating network of the microfluidic immunostaining system. Fluorescein isothiocyanate (FITC) solution was introduced into the inlet with distilled water. (**D**) A SKBR3 cell section was stained with graded concentrations of HER2 Ab and labeled with QD605 (left) and 3,3′-diaminobenzidine (right).

A new microfluidic linear concentration gradient generator was designed for the negative pressure-based operation of the microfluidic system (Fig. 1B). Compared to the conventional Christmas tree gradient generator^27^, the new generator has one more branch for each concentration inside the fluidic network. This additional branch channel for each concentration creates a linear profile of the concentration gradient at the end of the fluidic network. Nine channels were designed for the generation of eight concentrations of Abs. Negative pressure was applied to the outlet of the device and the Ab–Ag reaction occurred for the selected incubation time^28^. Prior to immunostaining, the concentration gradient generation was validated with fluorescein isothiocyanate (FITC) and distilled water (Fig. 1C). A linear gradient was generated using conventional microfluidic techniques requiring complicated chip designs or two additional pumps^29^,^30^,^31^. Compared to conventional concentration generators that use positive pumping through the inlet, our negative pumping-based system could be more stable because random fluctuation from the micropump cannot theoretically make a flow rate difference between two inlets. This is because the microfluidic channel, including the microfluidic network, is symmetrical for each inlet based on the outlet. When the fluctuation appears at a conventional two-pump injection system, there could be a flow rate difference between two inlets because fluctuation appears at unexpected time points at each pump connected to the respective inlet, and it could destroy the concentration gradient. On the other hand, using the negative pumping, the same negative pressure is applied to each inlet. Although the flow rate of the whole microfluidic system can be changed due to fluctuation, however, the concentration gradient can be maintained because the fluctuation of two inlets can be synchronized with our system. Using our system, we verified that Ab incubation and staining were possible with both fluorescence and color reagents (Fig. 1D).

### Comparison of on-chip and batch immunostaining

**Figure 2:**
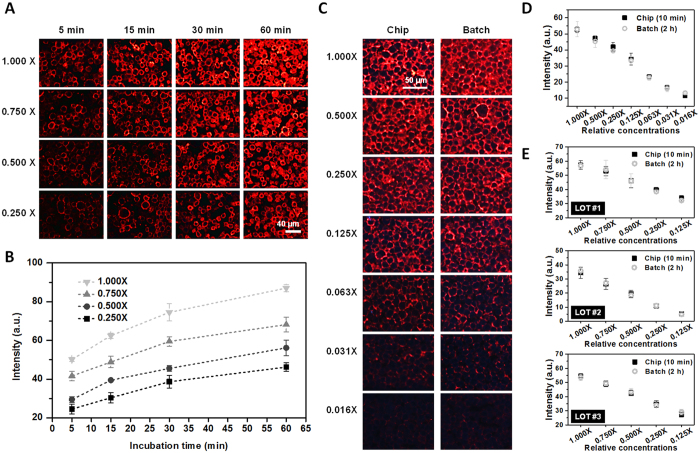
Comparison of the staining intensities between a batch process and a microfluidic process using HER2 Abs and QD605-IgG with SKBR3 cell sections. (**A**,**B**) Staining results using the microfluidic device at 5, 15, 30, and 60 min. The data are mean ± s.d.; *n* = 3–4. (**C**) HER2 Ab was labeled with QD605 using a microfluidic immunostaining system (10 min) and a batch staining process (2 h); the concentration ranged from 0.375 ng mL^−1^ to 24 ng mL^−1^. (**D**) Quantitative analysis of the HER2 staining intensity between a batch process and microfluidic staining. The data are mean ± s.d.; *n* = 3–7. (**E**) Staining intensities of three HER2 Abs (Dako: 1.6 μg mL^−1^, Abcam: 0.024 μg mL^−1^, Thermo Fisher Scientific: 0.36 mg mL^−1^ [for 1.000×]) at several concentrations using a 2 h batch process and a 10 min microfluidic process. The data are mean ± s.d.; *n* = 3–8 for 2 h batch process and *n* = 3–4 for microfluidic process.

The Ab–Ag reaction is accelerated due to effective mass transport when the Ab solution flows over an Ag-fixed substrate through a microfluidic channel^32^. Usually, the Ab incubation time in a pathology laboratory should be about 1 to 2 h for sufficient labeling of Abs on proteins. Accordingly, we tried to identify a fluidic immunostaining condition such that the result had the same staining intensity after 2 h of batch Ab incubation. To observe the Ab–Ag reaction in a microfluidic channel, HER2 Ab was incubated in SKBR3 cell sections using a microfluidic channel at several time points. The staining intensity was quantified using QD605 (emission peak: 605 nm) labeling and the ImageJ program (NIH, USA). The velocity of the Ab solution flow was set to 100 μm s^−1^ in the channel, which is the minimum velocity that the microfluidic system can operate, for accurate matching of the staining intensity between microfluidic staining and 2 h batch staining (because the flow-based Ab–Ag reaction in a microchannel is affected by the channel flow rate). Among eight concentrations, four were analyzed for the Ab staining tendency when a concentration of 1.000× was set to 1.7 μg mL^−1^ (Dako, Denmark). At all Ab concentrations, the trend of the staining intensity variation with incubation time was similar (Fig. 2A). As the incubation time increased, the slope of the staining intensity graph decreased for every concentration (Fig. 2B). The amount of labeled HER2 Ab on the protein increased proportionally with incubation time, up to 30 min, using the microfluidic device.

SKBR3 cell sections were immunostained with HER2 Ab (Abcam, USA) at a range of 0.375 ng mL^−1^ to 24 ng mL^−1^ using a 2 h batch incubation method (Fig. 2C: 1.000× for 24 ng mL^−1^). The staining intensities of 2 h batch Ab incubation were most similar to those obtained after 10 min Ab incubation using the microfluidic device (Fig. 2C,D). SKBR3 cell sections were immunostained with HER2 Abs produced by three different manufacturers (Dako, Abcam, and Thermo Fisher Scientific [USA]) using a 2 h batch incubation method at five concentrations (0.125×, 0.250×, 0.500×, 0.750×, and 1.000×). All of the 1.000× concentrations were assigned to random values based on the batch Ab staining results, the recommended values from the manufacturers, and the subjective analysis of intensities matching the staining intensities of three HER2 Abs (Dako: 1.6 μg mL^−1^, Abcam: 0.024 μg mL^−1^, Thermo Fisher Scientific: 0.36 mg mL^−1^). We randomly labeled the HER2 Abs (LOTs #1, #2, and #3) to preserve anonymity.

All of the staining results obtained using the microfluidic device were similar to those of the 2 h batch Ab incubation (Fig. 2E). At all concentrations under the Ab incubation, the differences in the average staining intensity between the microfluidic chip process and the batch process were less than approximately 4%, except for the 0.125× concentration of LOT #3 Ab (6.55%). This means that the screening of different Ab incubation conditions with the microfluidic chip can be used to predict the batch 2 h incubation results at various concentrations of Ab.

### Standardization of HER2 staining intensity

**Figure 3:**
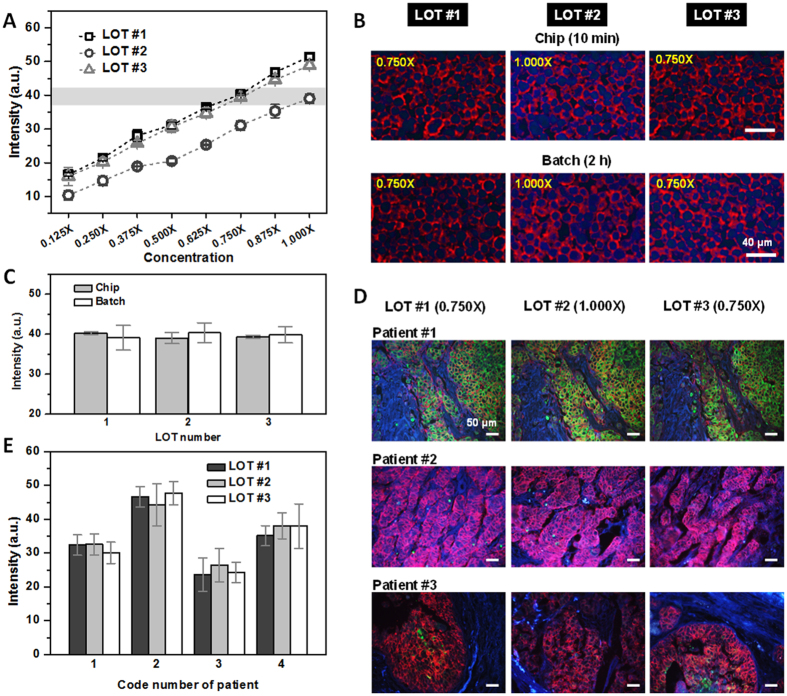
Standardization of three HER2 Ab immunostaining and standardized tissue staining results. Three HER2 Abs from different companies (Dako: 1.6 μg mL^−1^, Abcam: 0.024 μg mL^−1^, Thermo Fisher Scientific: 0.36 mg mL^−1^ [for 1.000×]) were randomly labeled as LOTs #1, #2, and, #3, respectively, to ensure anonymity. The chip-based Ab-staining process was carried out for 10 min and the batch process was carried out for 2 h. (**A–C**) Based on the 1.000× intensity of LOT #2, 0.750× of LOTs #1 and #3 have the most similar intensity. The data are mean ± s.d.; *n* = 3. Red color shows the QD605-labeled cells in tissue sections and blue color comes from the autofluorescence of tissue sections caused by the excitation of ultraviolet. (**D**) Sections from the same breast cancer tissues were immunostained with selected concentrations of HER2 Abs. (**E**) Intensity quantification result of tissue sections with HER2 Abs. The data are mean ± s.d.; *n* = 3–15. The error bars are based on the QD605 intensity of the imaging spot only from the epithelial region in the same tissue section.

With this system, we standardized the staining intensity of three HER2 Abs (Dako: 1.6 μg mL^−1^, Abcam: 0.024 μg mL^−1^, Thermo Fisher Scientific: 0.36 mg mL^−1^) using the microfluidic system (Fig. 3). After selecting concentrations that resulted in the best staining intensity matched for the three Abs (gray region in Fig. 3A: 0.750× for LOTs #1 and #3, 1.000× for LOT #2), a 2 h batch staining was performed with the selected concentrations of Abs (Fig. 3B,C). From the fluidic network, eight concentrations (0.125×, 0.250×, 0.375×, 0.500×, 0.625×, 0.750×, 0.875×, and 1.000×) were created, and simple criteria were established to select the most similar values, using threshold values for each concentration, based on the midpoint of the intensity difference between adjacent concentrations.


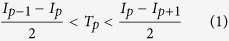


where *p* is the selected concentration point, *p−1* and *p* + *1* are adjacent concentration points (e.g., if *p* is selected as 0.500×, then *p−1* is 0.375× and *p* + *1* is 0.625×). *I*_*p*_ is the numerical intensity value at *p*, and *T*_*p*_ is the threshold value at *p*. The average values of the intensity gap between the concentration points (*I*_*p*_*−I*_*p−1*_) were as follows: LOT #1: 4.98 ± 1.32, LOT #2: 4.09 ± 1.28, and LOT #3: 4.70 ± 0.56 (Fig. 3A). In the case of LOT#1, one concentration point covers 4.98 ± 1.32 intensity values; thus, the maximum possible error will be 4.98 ± 1.32 at every concentration. Obviously, the percentage of this value to staining intensity increases as concentration decreases. For example, the percentage of *T*_*p*_ to staining intensity at 1.000× of LOT #1 is 4.85%, while that at 0.125× of LOT #1 is 15.09%. Therefore, 1.000× intensity of LOT #2 was selected as a reference intensity to reduce errors.

The average staining intensities using a conventional 2 h batch process with HER2 Abs of LOTs #1, #2, and #3 were 39.11 ± 3.12, 40.34 ± 2.48, and 39.87 ± 2.03, respectively (Fig. 3C). In this case, the difference in the average intensity among three Abs was less than 1% of any average intensity of those three Abs. Only 10 min, 5 μL of Ab solution, and a single reference slide were needed to predict the 2 h HER2 Ab staining results of 24 SKBR3 cell section slides. The primary Ab incubation time decreased despite the fixing of the flow velocity of our device to its minimum velocity of approximately 100 μm s^−1^. The device operation time can thus be further reduced, by increasing the flow velocity or by applying another optimization process.

To verify the performance of our system in tissue biomarker analysis, the selected concentrations of HER2 Abs (0.750× for LOTs #1 and #3, 1.000× for LOT #2) were used for the immunostaining of real human tissue sections from breast cancer patients. HER2 over-expressing tissues (score of 3+) were selected by pathologists to demonstrate our system. Three section slides from the same tissue block were immunostained with three HER2 Abs at selected concentrations using a conventional 2 h batch process. The selected tissue sections were arranged adjacent to each other before sampling, to minimize the staining intensity variation due to tissue heterogeneity. Fluorescence images of the same locations in tissue sections were acquired to compare the HER2 staining intensity. To facilitate the finding of the same locations in the tissue, a double color QD staining was performed to stain cytokeratin and HER2, to mark the epithelial region of a tissue and simultaneously target the biomarker (Fig. 3D)^33^. Due to the cell heterogeneity of the breast cancer tissue^34^, the difference in the average QD intensities among the HER2-labeled tissue sections was larger than that among the cell sections (Fig. 3E). The maximum difference in the average intensity between the tissue sections, stained by LOTs #1, #2, and #3 from the same tissue block, did not exceed 11.69% of any average intensity of LOTs #1, #2, or #3 (6.98%–11.69%) (Fig. 3E).

### Ab quality verification using a microfluidic system

**Figure 4:**
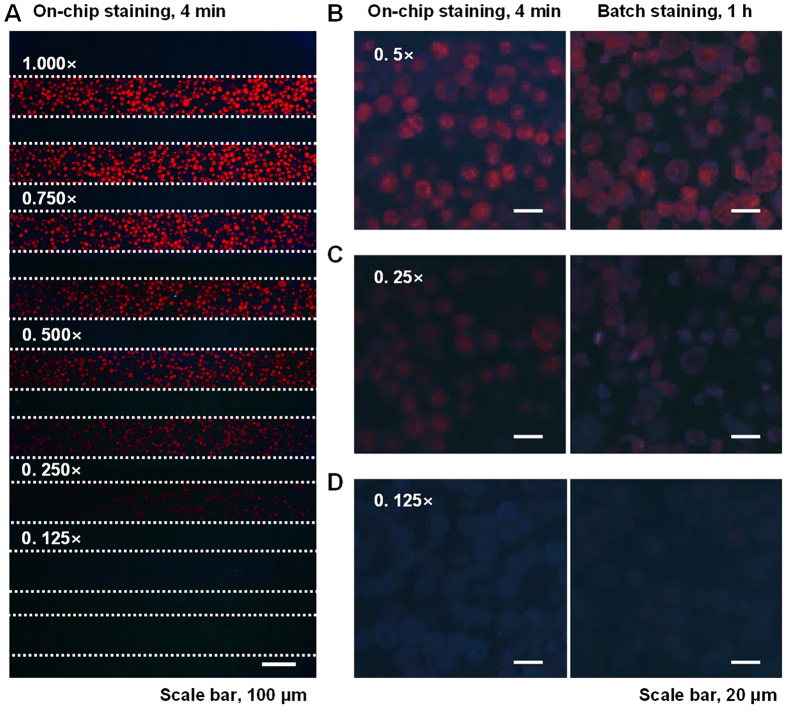
Fluorescence images of QD605s in SKBR3 cell sections labeled with a form of quantum dot-conjugated IgG (QD-IgG). (**A**) Fluorescence images of QD605-conjugated goat anti-rabbit IgG incubated in a SKBR3 cell section using a microfluidic immunostaining system. (**B–D**) Comparison of fluorescence images stained using a microfluidic system (left) and a batch process (right). The incubation results from concentrations of 0.5×, 0.250×, and 0.125×. Red color shows the QD605-labeled cells and blue color comes from the autofluorescence of cell sections caused by the excitation of ultraviolet.

Secondary Ab has been widely used in IHC due to its flexibility and efficiency. We demonstrated that our microfluidic immunostaining system can be used as a predictor of the non-specific binding conditions of the secondary Ab incubation. The non-specific binding of QD605-conjugated goat anti-rabbit Ab (Thermo Fisher Scientific) was demonstrated by the incubation of QD-IgG in SKBR3 cell sections using a microfluidic system and a conventional batch process (Fig. 4). The cell section was incubated with QD-IgGs concentrations from 0.250× to 1.000×. The signals of QDs were not detected under 0.125× QD-IgG concentration with our analysis system (Fig. 4A). Conventional batch QD-IgG incubation was performed with 0.125×, 0.25×, and 0.5× QD-IgG concentrations, and the immunostaining results were the same as those from the microfluidic system (Fig. 4B). This means that it is possible to find an incubation condition resulting in maximum biomarker signals, but a minimum non-specific binding of QD-IgG.

**Figure 5:**
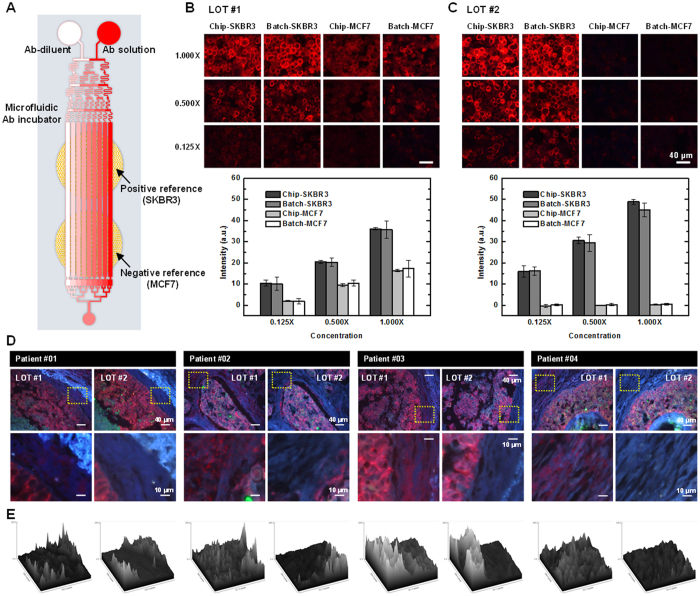
Concept of the Ab quality verification system and fluorescence images of breast cancer cells and breast cancer tissues. (**A**) A schematic of the microfluidic immunostaining system and a cell array consisting of SKBR3 and MCF7. SKBR3 and MCF7 were used as positive and negative references for HER2, respectively. (**B**) Immunostaining results of HER2 Ab: LOT #1 in SKBR3 and MCF7 using a microfluidic system and batch incubation process. The data are mean ± s.d.; *n* = 3 for chip and *n* = 3–9 for batch. (**C**) HER2 Ab: LOT #2 was used as in panel B. (**D**) Immunostaining results of breast cancer tissues from four different patients using HER2 Abs of LOT#1 and LOT #2. (**E**) Surface plots based on the intensities of QD605; bottom images of panel D.

To demonstrate the HER2 Ab quality testing in our system, a cell block containing both positive and negative HER2 references was fabricated (Fig. 5). Positive and negative reference cell sections were fabricated as a linear microarray so that microfluidic Ab incubation channels could cover both positive and negative reference cells (Fig. 5A). Two HER2 Abs were compared with this experimental setting. The HER2 Ab staining results from the LOT #1 incubation show that QD signals were detected in both SKBR3 and MCF7. The QD intensities at SKBR3 and MCF7 increased as the Ab concentration was increased (Fig. 5B). However, the QD intensity at MCF7 did not increase in the LOT #2 HER2 Ab staining results as the Ab concentration was increased (Fig. 5C). Additionally, the QD intensity was approximately zero. Conventional batch Ab incubation was also processed at concentrations of 0.125×, 0.500×, and 1.000×. As we found in previous experiments, immunostaining results between the microfluidic system and the batch process were similar (Fig. 5B,C, graphs). SKBR3 and MCF7 cells are known to be HER2 positive and negative, respectively^35^. If the QD signal detected from MCF7 represents the non-specific HER2 signal, then the non-specific intensity percentage of the positive reference (in this case, the QD intensity detected from SKBR2) should be as follows: 0.125×: 18.13%, 0.500×: 51.23%, and 1.000×: 48.45%. In contrast, LOT #2 HER2 had percentages as follows: 0.125×: 3.23%, 0.500×: 1.64%, and 1.000×: 0.93% (Fig. 5B,C). We can infer that LOT #2 HER2 Ab is superior to that of LOT #1 only with our specificity criteria.

To verify the quality difference between two HER2 Abs, breast cancer tissue sections from four different blocks were immunostained with conventional batch process using 1.000× concentrations in both LOT #1 and LOT #2. Fluorescence images shows that the QD signal was detected at both cell regions and non-cell regions of tissues labeled with LOT #1 Abs: the QD signal was not detected in non-cell regions in tissues labeled with LOT #2 (Fig. 5D). A surface plot of the QD605 signal showed that LOT #2 Ab more clearly distinguishes HER2-expressing cell regions and non-cell regions than LOT #1 Ab (Fig. 5E).

## Discussion

Following the initial development of the IHC, quality verification and assurance of Abs were subjective processes due to the lack of adequate technology. Here, we presented a new IHC quality assurance system for the standardized cancer biomarker analysis using microfluidic technology. We only controlled the Ab staining concentrations for the standardization of staining intensity and fixed other factors that affect the staining intensity, such as temperature and incubation period, to avoid the complexity of the standardization process. The intensity of the 2 h batch immunostaining can be predicted using our microfluidic approach in various concentrations with a process time of 10 min. The 2 h batch Ab staining results showed that similar intensities can be reproduced in both cell sections and human breast cancer tissues with different HER2 Abs. The whole tissue IHC system, developed by Ciftlik *et al*., was used to quickly and accurately determine HER2 status^36^. This kind of whole tissue IHC system can provide a better repeatability of the IHC assay than any other method of IHC. The standardization of Ab staining will be more effective when this system is used along with our microfluidic Ab staining system.

Ab incubation condition was identified that resulted in a maximum biomarker signal but minimum non-specific binding of QD-IgG. Quality verification of HER2 Ab was performed using a cell slide containing both positive and negative references of HER2. The IHC results showed that HER2 Ab quality verification using our system reflects IHC staining quality in human breast tissue. There are also many breast cancer cell lines that can be used as positive and negative references in HER2 quality tests. Because the reference slide fabrication method is the same as the conventional tissue microarray (TMA) fabrication method, combining cells or screening multiple cells could be more effective for verifying Ab quality. Our microfluidic system can also be integrated with TMA for enhanced efficiency in any IHC tests^23^.

Although the tissue staining analysis was processed qualitatively, staining results from the negative reference (MCF7) using our system can predict a non-specific background signal in tissue staining results. This means that our method of Ab quality testing can be used in pathology laboratories or hospitals to verify Ab quality. Although we have demonstrated our method with a microfluidic device that produces eight graded concentrations of Ab, it is possible to increase the number of channels by expanding the microfluidic network because our design has a regular pattern (Fig. 1B). Increasing the number of channels will lead to enhanced accuracy.

The use of protein-coated substrate, such as a protein array, with our microfluidic immunostaining device will yield more accurate comparisons of Ab staining intensity than the use of cell section slides, which consist of a cell line such as SKBR3^37^. However, the use of a protein array affords no opportunity to employ qualitative Ab labeling analysis, such as protein localization, which is correlated with the identification of the non-specific binding of Abs at a cellular level. Furthermore, the long-term storage of protein-coated substrate using recent technologies is difficult, which can lead to an additional quality assurance problem. We verified our system with QD-IgG due to its superior optical properties, to facilitate quantification and to increase sensitivity. However, conventional color reagents, such as 3,3′-diaminobenzidine, can also be used with our system.

In summary, we confirmed that it is possible to quantitatively equalize the immunostaining quality of three different HER2 antibodies using a microfluidic staining intensity matching system. Our system helped us to find an optimized immunostaining condition that ensured a maximum biomarker signal, but a minimum background signal. Considering that the most critical issue in the IHC is quality assurance, this approach is of great importance as a benchmark for the quality assurance and standardization of the future cancer diagnostic testing.

## Methods

### Preparation of cell, cell array, and tissue sections

SKBR3 and MCF7 cells were purchased from ATCC and cultured in RPMI-1640 supplemented with 10% fetal bovine serum (FBS), 100 IU mL^−1^ penicillin, and 100 mg mL^−1^ streptomycin. All cells were maintained at 37 °C and 5% CO_2_. The cell lines were authenticated by examination of morphology and growth characteristics for the confirmation of mycoplasma free. For the creation of cell paraffin block, cultivated cells were detached from the cell culture dishes after trypsinization. Harvested cells were then centrifuged at 1500 rpm for 5 min. Agar suspension, formalin fixation, and paraffin embedding processes were performed to make a cell block. Paraffin embedded blocks were then sectioned at 3 μm, the sections were mounted and baked onto positively charged glass slides. These samples were dried for 1 h at room temperature followed by 1 h in an incubator at 65 °C. For the preparation of cell array, SKBR3 cell paraffin blocks were punched with a 2 mm-diameter tissue microarray punch. The obtained 2 mm diameter cell blocks were then inserted to the blank paraffin block for the fabrication of the cell array. The gap between cell blocks in blank paraffin was 1 mm to follow the standard of commercial tissue microarrays. This cell array block was sectioned at 3 μm, mounted on glass slides, and dried for 1 h at room temperature to make section slides.

Human tissue samples were obtained from the National Cancer Center (Goyang, Korea), with the corresponding written consent provided by the patients or their relatives. This study was approved by the Institutional Review Boards at the National Cancer Center (NCCNCS-12-648) and qualified exemption under the Korea Advanced Institute of Science and Technology. The tissue samples were fixed for 24 h in 4% neutral-buffered formalin, Bouin’s fixative, acetic formalin alcohol, or 4% or 10% unbuffered formalin, 4 h in PreFer (Anatech; Battle Creek, MI) or Pen-Fix (Richard Allen Scientific; Kalamazoo, MI), or 48 h in 4% neutral-buffered formalin. When the paraffin embedding procedure was completed, tumor specimens were cut into 4-μm sections, dried for 1 h at room temperature, and then incubated for 1 h at 60 °C. All tissues were scored as HER2 3+ by pathologists at the National Cancer Center.

All experiments in this study were controlled in compliance with ethical and safe research practices involving human subjects or tissues. No informed consent was required because the samples were analyzed anonymously and no identifying information relating to participants were included.

### Materials for immunostaining

HER2 Abs from three Ab producing companies (Abcam: ab134182, Thermo Fisher Scientific: PA5-16305, and Dako: A0485) were used for the demonstration of our system. All HER2 Abs were originated from rabbit. Mouse monoclonal cytokeratin Ab was used to determine the epithelial region of the tissue (Dako: M3515). QD605 goat anti-rabbit IgG (QD605-IgG, Thermo Fisher Scientific: Q-11401MP) and QD525 goat anti-mouse IgG (QD525-IgG, Thermo Fisher Scientific: Q-11041MP) were exploited to label cytokeratin Ab with QD525 and to label HER2 Ab with QD605. 1× for QD605-IgG and QD502-IgG is 1:50 dilution condition (20 nM). TRIS buffered saline plus Tween 20 (TBS-T, Scy Tek: TBT999) was used as a washing buffer. 2% BSA/5% goat serum/PBS was used as a blocking solution. The target retrieval solution (Dako: S1699) was used to conduct a microwave antigen-retrieval technique.

### Design and fabrication of the microfluidic device

The channels in the microfluidic network have a width of 60–96 μm and a height of 60 μm. For the efficient mixing of solutions at low Reynolds number, we placed ridges on the top of the channel at an oblique angle (height: 30 μm) by two-step lithography of SU8 photoresists on silicon wafer^38^. All channels in the microfluidic network have a length of more than 4 mm based on the simulation result, so that the complete mixing was accomplished at 4 mm from the beginning point of the mixing (data not shown). The immunoreaction channels have a width of 120 μm and a height of 60 μm.

### Immunostaining method for batch process and microfluidic process

The section slides were soaked in xylene for the removal of paraffin and rehydrated using a graded series of ethanol solutions. After hydration, a microwave-based Ag-retrieval was processed for 20 min in a target retrieval solution at pH 9.0 and 750 W. Following Ag-retrieval, the slides were treated in blocking solution to prevent non-specific binding of the secondary Abs. For the conventional batch incubation process, an Ab solution (200 μL) was applied to the section slide and incubated for 2 h. After washing the slide using TBS-T (three times for 1 min each), QD605-IgG was added and incubated for 1 h. To conduct a microfluidic process, the Ag-retrieved slide was soaked in TBS-T after the blocking process. A plasma-treated microfluidic device was soaked together with the section slide in TBS-T and attached on the section slide by aligning channels to cells. After mounting the microfluidic device on the section slide, the inlets were filled with an Ab diluent and a primary HER2 Ab solution, and a syringe pump was linked to the outlet of the device to induce a flow for the generation of concentration gradient over the surface of the cell section. This process needed 10 min and required 5 μL primary Ab solution. Like a conventional batch process, the QD-IgG incubation was performed after the detachment of the microfluidic device from the cell section slide and washing using TBS-T (three times for 1 min each). Only a primary Ab incubation process was performed using a microfluidic system. For the verification of our system concept, more than three cell sections were used for the microfluidic immunostaining, because cell blocks were made of relatively homogeneous cells.

### HER2 staining intensity quantification

A quantitative image process was carried out to quantify the QDs labeled on HER2 protein. The images were obtained by a charge-coupled camera (DP72; Olympus) equipped with a fluorescence microscope (IX72; Olympus). The ultraviolet excitation and the acquisition of the emission signal from the QDs were carried out using the same objective lens. After the acquisition of the fluorescence images, the QD605 signal was separated from the original image and the signal was numerically quantified by the ImageJ program (NIH). All imaging parameters and equipment were fixed and maintained at a certain condition, and numerical intensities of images were obtained at the same imaging condition for each experiment set, such as the exposure time for the quantitative comparison of the staining results. For the quantification of QDs on cell sections, which were stained using a microfluidic device, at least three images were obtained from each cell section using a 20× objective lens. On the other hand, three to nine images were obtained from the cell sections stained by the batch process. The sample size was dependent on the dimension of the microfluidic channel and the cell section. Experimental data were excluded that were physically damaged by handling mistakes, or results from microfluidic channel failure during immunostaining.

### Statistical analysis

Numerical mean values of the QD605 staining intensity were calculated by the ImageJ analysis program with threshold function, thereby eliminating the background signal of the samples. One image contains about 400 cells per cell block sample. At least three cell block section samples were analyzed to obtain the average values and standard deviation for error bars. The threshold values were fixed at a specific point for cell and tissue samples for the quantitative comparison.

### Data availability

The data that support the findings of this study are available from the corresponding author on request.

## Additional Information

**How to cite this article:** Kwon, S. *et al*. A Microfluidic Immunostaining System Enables Quality Assured and Standardized Immunohistochemical Biomarker Analysis. *Sci. Rep.* **7**, 45968; doi: 10.1038/srep45968 (2017).

**Publisher's note:** Springer Nature remains neutral with regard to jurisdictional claims in published maps and institutional affiliations.
